# Recurrent achalasia after Heller-Toupet procedure: Laparoscopic extended redo heller myotomy and floppy Dor

**DOI:** 10.4103/0972-9941.37193

**Published:** 2007

**Authors:** Vishwanath Golash

**Affiliations:** Department of Surgery, Sultan Qaboos Hospital, P.O. Box: 98, Salalah, 211, Sultanate of Oman

**Keywords:** Achalasia cardia, Heller-Dor, laparoscopy, redo

## Abstract

Recurrences of symptoms after the surgery for achalasia cardia are not uncommon. There are several causes of recurrences but the early recurrences are speculated to be secondary to incomplete myotomy and late recurrence due to fibrosis after the myotomy or megaesophagus. These recurrences can be managed by regular dilation failing which a redo surgery is indicated. Laparoscopic approach is now standard because of the obvious benefits for patients and surgeons. Extent of myotomy and addition of fundoplication are debatable issue in the management of achalasia cardia but evidence suggests that some kind of fundoplication would be necessary after the complete division of lower esophageal sphincter. We present our experience in a case of recurrent achalasia, secondary to incomplete myotomy managed laparoscopically by extended myotomy and a floppy anterior fundoplication. Patient is asymptomatic six months after the surgery and radiologically there is free passage of barium in the stomach.

## INTRODUCTION

Achalasia cardia is a disease of unknown etiology characterized by aperistaltic esophagus and increased lower esophageal sphincter pressure. The esophagocardiomyotomy is the most effective palliative surgical treatment but there is a substantial recurrence rate after surgery. We present a case of recurrent achalasia cardia managed successfully by laparoscopic redo extended esophagocardiomyotomy and anterior fundoplication.

## CASE REPORT

A 60-year-old man presented with the history of progressive dysphagia, regurgitation and an offensive smell of fermented food from his mouth since his surgery for achalasia cardia 32 years ago. The precise details of the surgery were not available. It was also not known whether an anti-reflux procedure was done at the previous surgery although a recent CT scan had shown a partial wrap. He claims that his dysphagia and regurgitation were immediately improved after his first operation. But after a year or so he started having dysphagia again which got progressively worse over the years. On examination, his general health was satisfactory. The previous Heller was done by open transabdominal approach and he had an upper midline laparotomy scar [Figure 1A]. His routine work-up revealed the following findings:

**Figure 1A:**
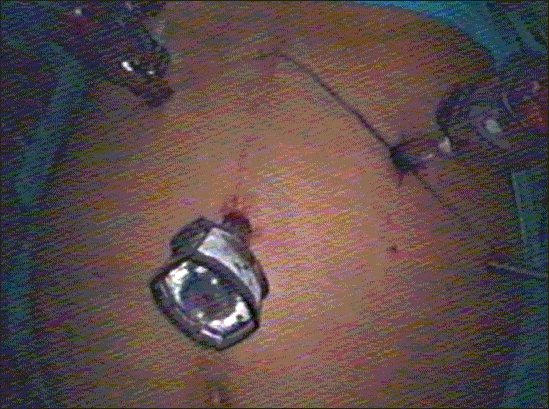
Port position and scar of previous surgery

**Endoscopy:** The esophagus was dilated and contained fluid residue, wall was hypertrophied with multiple contraction ring. There was no stricture at the lower end and scope passed easily through to the stomach.

**Barium meal:** A persistent bird's beak image of cardia with a dilated sigmoid esophagus [Figure 1B].

**Figure 1B:**
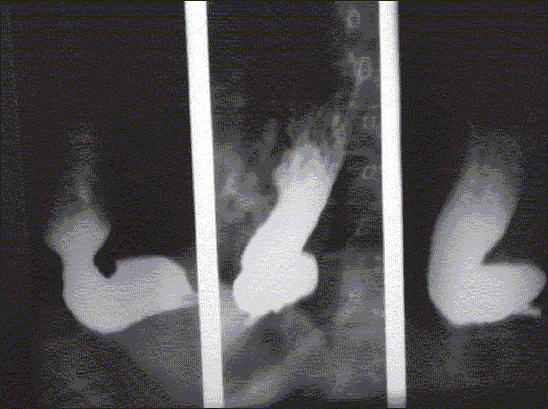
Barium meal

CT chest and abdomen: Dilatation of esophagus with air fluid level seen up to gastroesophageal junction. Dilatation at its maximum was 7.8 cm (grade IV). Below the diaphragm a small paragastric air fluid level was seen due to previous surgery [Figure 1C].

**Figure 1C:**
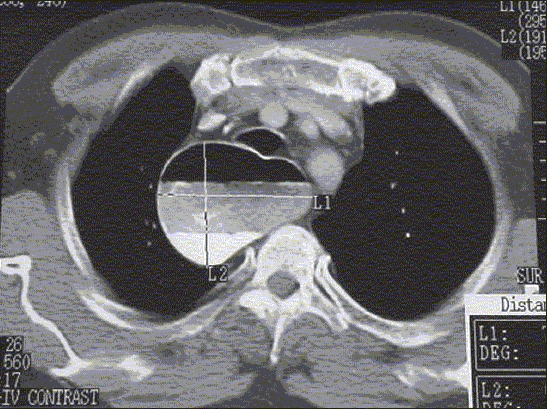
CT scan chest and abdomen

Unfortunately, the pH and manometry services were not available to study the lower esophageal sphincter pressure and the reflux. The radiological and endoscopic findings were highly suggestive of recurrent achalasia cardia. He underwent three courses of progressive balloon dilatation but showed no improvement in his symptoms and was referred for surgery.

The port positions were same as for laparoscopic hiatus hernia repair [Figure 1A]. The liver was completely adherent to the diaphragm and retraction of liver was not necessary requiring only four ports [Figure 1D]. The surgery was difficult because of loss of planes, distorted anatomy, excessive scarring and severe adhesions in the esophagogastric region. After adhesiolysis around the hiatus area, it became clear that a posterior fundoplication was attempted at previous surgery, which had disrupted. We chipped away slowly and steadily and the hiatus and the crure were cleared. The esophagus was mobilized circumferentially in the posterior mediastinum and was straightened. The posterior vagus nerve was identified after undoing the fundoplication, the anterior vagus nerve was seen proximally in the mediastinum to the left of the previous myotomy and was lifted away to complete the myotomy, excessive care was taken not to injure the vagi. The proximal myotomy was extended to 8-9 cm and the lower limit of the cardiomyotomy was extended up to 3 cm on the stomach to completely divide the lower esophageal sphincter.[1] Myotomy was performed using ultracision shears [Figure 2A, B]. Per-operative endoscopy was helpful during the surgery. Completeness of the myotomy was confirmed on table endoscopy and at the same time air leak test was done to rule out any mucosal perforation. The posterior fundoplication was converted to floppy Dor fundoplication after dividing the short gastric vessels [Figure 2C, D]. The Surgery lasted for 180 minutes. There were no per-operative complications and no blood transfusion required. He was fed liquids on first postoperative day. His dysphasia and regurgitation improved considerably and postoperative barium meal done at one month and six months showed the cardia diameter of more than 1 cm, significant reduction in the esophageal diameter and free passage of barium in the stomach.

**Figure 1D:**
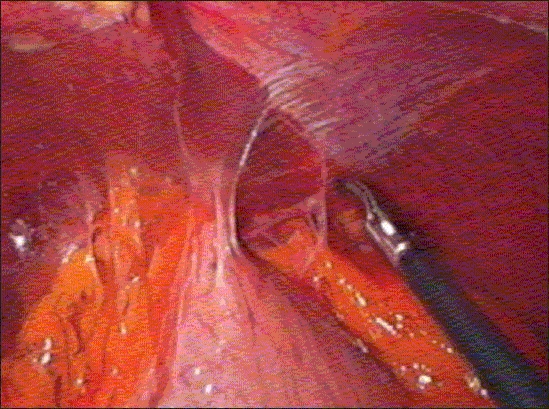
Laparoscopic view of hiatus area before surgery

**Figure 2A:**
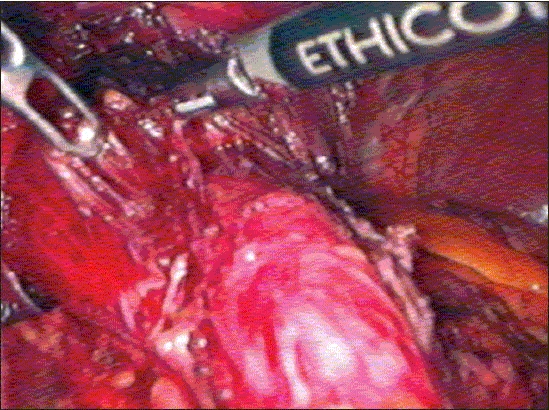
Myotomy with ultracision shears

**Figure 2B:**
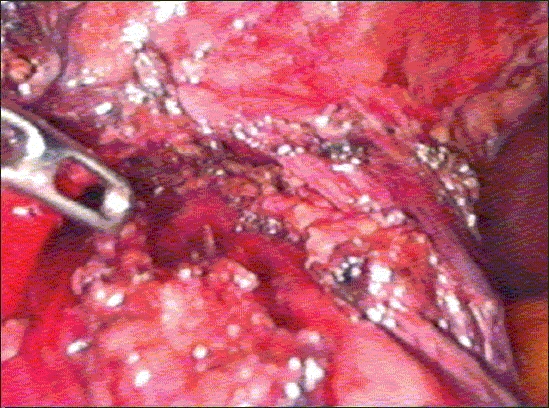
Extended myotomy

**Figure 2C:**
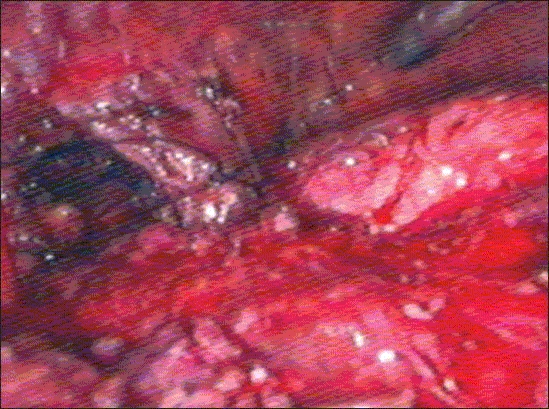
Fixing the fundus to left crura

**Figure 2D:**
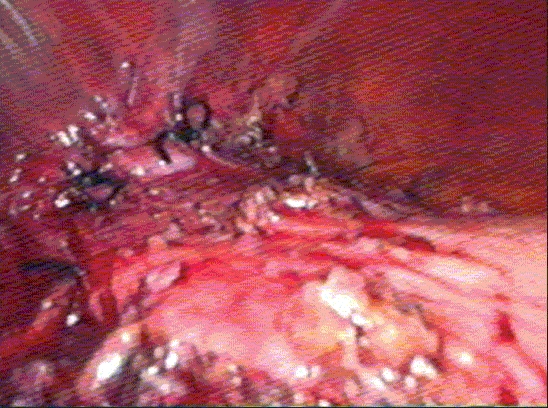
Completed Floppy Dor Fundoplication

## DISCUSSION

The common causes of recurrences are incomplete or inadequate myotomy, scarring, megaesophagus prior to surgery, constricting fundoplication and gastroesophageal reflux. It is recommended that the completeness of the myotomy should be checked intraoperatively by endoscopy and manometry when available. Extent of myotomy and addition of fundoplication are debatable issue in the management of achalasia cardia but evidence suggest that some kind of fundoplication would be necessary after the complete division of lower esophageal sphincter Heller -Dor gives better results than Heller myotomy alone.[2] Myotomy is still appropriate in patients with megaesophagus and surgery should not be limited to less severe morphological types, especially by comparison with the alternative options which is esophagectomy.[3]

Although the myotomy can be performed by electrodiathermy or ultracision shears, but the ultracision was particularly useful in redo surgery to have a bloodless, smokeless field and hastens the surgery. But great care and experience is required in its use as the back end of shear can damage the mucosa. The comfort of working in a bloodless field and taking less time negates the cost.[4,5]

Other debatable issues in this difficult scenario are the use of an alternative cavity (thoracic) free of adhesions and using posterior myotomy instead of anterior. The mobilization of the esophagus in the mediastinum, an anti-reflux procedure, the ergonomic of operating in the axis of esophagus and extension of myotomy in either direction is easily done laparoscopically. A thoracic/thoracoscopic approach is cumbersome, requires a double lumen tube for anesthesia, lateral decubitus position, difficult ergonomic position to access the esophagus, postoperative chest drain, greater postoperative pain, longer operative time, longer hospital stay and a higher incidence of persistent dysphagia and secondary gastroesophageal reflux postoperatively.[6] Surgeon used to thoracic approach have their argument, since the phrenoesophageal ligament is not divided and anti-reflux procedure is not required. The thoracoscopic approach would have been difficult in this patient not knowing the details of previous surgery, a hugely dilated sigmoid megaesophagus in the chest and danger of injury to other viscera adherent to gastroesophageal region.

Although Ernst Heller in his original technique described the two cardiomyotomy techniques, one anterior and one posterior, this operation was subsequently modified to only anterior incision (and now laparoscopically). After dismantling the previous posterior fundoplication, performing a posterior myotomy was another possibility but there is paucity of experience and results after posterior myotomy in published literature.

Redo Hellers is technically challenging operation especially in this particular patients where surgery was done several years ago and details were not available. It is a major operation and demand experience in advance laparoscopic surgery of the esophago-gastric region. Excessive care is required in protecting the vagi, avoiding injury to mucosa and to confirm the completeness of myotomy on table. Endoscopic dilatation may be still being required at intervals in some patients after myotomy for the sclerosis of cardia.
